# Comparative Analysis of Pain Perception, Corticomotor Excitability, Disability, and Cognitive Function in Chronic Low Back Pain Patients and Age-Matched Healthy Controls

**DOI:** 10.7759/cureus.93103

**Published:** 2025-09-24

**Authors:** Santosh L Wakode, Rahul Gour, Shasidharan Krishnan, Vithal Puri, Ankur Wakode, Avinash Thakare, Sunil Chouhan, Sandip Hulke

**Affiliations:** 1 Physiology, All India Institute of Medical Sciences, Bhopal, Bhopal, IND; 2 Physical Medicine and Rehabilitation, All India Institute of Medical Sciences, Bhopal, Bhopal, IND; 3 Physiology, All India Institute of Medical Sciences, Nagpur, Nagpur, IND

**Keywords:** chronic non-specific low-back pain, motor evoked potential, neuroplasticity, p 300, repetitive transcranial magnetic stimulation (rtms), resting motor threshold

## Abstract

Background and introduction

Chronic low back pain (CLBP) is a common, disabling condition with a multifactorial aetiology that extends beyond nociceptive dysfunction. Among the Indian population, low backache is a significant contributor to years lived with disability (YLD). Recent evidence suggests that CLBP can cause significant alterations in central nervous system functioning and neuroplasticity, via deficits in intracortical modulation involving glutamatergic and gamma-aminobutyric acid (GABA)-mediated mechanisms. Understanding the broader impact of CLBP and its effects on the nervous system - particularly the motor system and cognition - is essential for developing comprehensive treatment strategies and evolving appropriate diagnostic and prognostic tools that extend beyond pain management.

Objective

The main objective of this study is to compare pain perception, disability, corticomotor excitability, and cognitive functions between patients with CLBP and age-matched healthy controls, to explore central nervous system alterations associated with CLBP.

Methodology

A total of 116 participants were enrolled, including 58 CLBP patients and 58 age- and gender-matched healthy controls. Pain intensity and disability were assessed using the Visual Analogue Scale (VAS) and the Modified Oswestry Disability Index (MODI), respectively. Corticomotor excitability was assessed using transcranial magnetic stimulation (TMS), which measured the resting motor threshold (RMT%) as a percentage of maximum stimulator output, and the motor evoked potential (MEP) amplitude in microvolts (µV). Cognitive function was assessed using the Self-Administered Gero-cognitive Examination (SAGE) score and event-related potential (ERP) P300. The P300 was measured for its latency (in milliseconds) and amplitude (in microvolts). The Mann-Whitney U test was used for statistical comparison between groups.

Results

CLBP patients showed significantly higher RMT% (median = 55%, interquartile range (IQR) = 15) and lower MEP amplitude (median = 144 µV, IQR = 218.75) compared to controls (median = 50%, IQR = 5, and median = 294.5 µV, IQR = 287) (p < 0.001). VAS and MODI scores were markedly elevated in the CLBP group (median = 6, IQR = 2, and median = 11.5, IQR = 9) versus 0 in controls (p < 0.001). Cognitive performance, as measured by SAGE, was significantly lower in patients with CLBP (median = 17, IQR = 4) than in controls (median = 19.5, IQR = 3) (p < 0.001). ERP results revealed prolonged P300 latency (median = 341 ms, IQR = 33.75) versus (median = 290 ms, IQR = 35.75) and reduced amplitude (median = 7.3 µV, IQR = 5.67) versus (median = 12.45 µV, IQR = 6.77) in the CLBP group (p < 0.001), as compared to healthy controls.

Conclusion

CLBP is associated with alterations in central nervous system functioning, with significant changes observed in corticomotor excitability, increased disability, and decreased cognitive function, along with delayed and diminished neurophysiological responses. A comprehensive, multidisciplinary treatment approach targeting both peripheral and central mechanisms - including neurocognitive rehabilitation - is required for chronic pain management.

## Introduction

Chronic low back pain (CLBP) is the most common musculoskeletal disorder, with a high prevalence and morbidity (approximately 619 million cases of CLBP worldwide), according to World Health Organization (WHO) data. CLBP is the leading cause of years lived with disability (YLD), affecting individuals across age groups, socio-economic strata, and geographical regions. About 8% of YLD in India is due to chronic low back ache, and 4.6% of disability-adjusted life years (DALY) is because of musculoskeletal disorders [1]. Changing lifestyles, rapid urbanization, and limited access to early diagnosis and treatment are the main reasons for the rapidly increasing burden of CLBP.

Traditionally, CLBP was viewed more as a disease of anatomical abnormalities, such as disc degeneration, spinal stenosis, or musculoskeletal injuries; however, the concept has evolved over the past century. Earlier, diagnosis depended on physical examination and radiological imaging, and treatment centred on rest, analgesics, and surgical management. However, the chronic nature of the pain, and its lack of correlation with structural findings, suggest that this approach is incorrect and ineffective in detecting the actual problem.

The variable symptomatology and multifactorial aetiology, with its chronicity and complexity, make it essential to have a multidisciplinary approach in evaluating and managing this chronic, disabling condition. CLBP can cause significant alterations in central nervous system functioning, such as changes in corticomotor excitability, cognitive processing, and neuroplasticity, via deficits in intracortical modulation involving glutamatergic and gamma-aminobutyric acid (GABA)-mediated mechanisms [2,3]. Understanding the interplay between pain perception, neural mechanisms, and cognitive function is essential for improving clinical outcomes and guiding evidence-based interventions.

Pain, in the present understanding based on the biopsychosocial model, is an interplay between biological, psychological, and social factors. The pathophysiology of chronic pain is predominantly contributed to by central nervous system sensitization, altered motor control, and cognitive-emotional components [4]. Altered activity in the brain's default mode network and structural changes in cortical areas, overactivation of microglia, and neuroinflammation may directly impact cognitive function in chronic pain [5-7]. Transcranial magnetic stimulation (TMS) offers advanced tools in neurophysiological research, such as motor evoked potentials (MEPs) and resting motor thresholds (RMTs), thus enabling the assessment of corticomotor excitability and neuroplastic changes in CLBP patients [8].

The interrelationship between pain, disability, corticomotor excitability, and cognitive impairment in CLBP is not well understood, as most earlier studies have examined these factors separately [8,9]. A lack of normative data and standardized neurophysiological protocols further limits progress. Novel therapies, like non-invasive brain stimulation, show potential but require clearer insight into the underlying pathophysiological mechanisms. Few studies have concurrently assessed corticomotor excitability and cognition in CLBP, leaving a major research gap.

The Self-Administered Gero-cognitive Examination (SAGE) [10] and event-related potentials (ERPs) - particularly P300 - were used as valuable indicators of cognitive function and processing speed, domains increasingly recognized as affected in chronic pain syndromes. This study compares CLBP patients and age-matched controls using clinical scales (Visual Analogue Scale (VAS) [7,11] and Modified Oswestry Disability Index (MODI) [12,13]) to comprehensively understand functional impairment, and neurophysiological measures (RMT and MEPs) to assess cortical excitability. This research will contribute novel insights into the extent and interrelation of motor cortex excitability and cognitive changes in CLBP, thereby justifying the urgent need for integrative diagnostic and rehabilitative approaches.

## Materials and methods

Study design

Analytical Cross-Sectional Study

After obtaining written informed consent, a total of 116 participants were included in the study and divided into two groups: 58 cases of CLBP and 58 age-matched healthy controls. Data were recorded at a single time-point assessment.

The study was carried out in the Neurophysiology (rTMS Division) Laboratory, Department of Physiology, All India Institute of Medical Sciences (AIIMS), Bhopal, India.

Sample size calculation

To ensure sufficient power to detect statistically significant differences between the groups, and assuming a medium effect size (Cohen's d = 0.55), the sample size was calculated using G*Power software (Heinrich-Heine-Universität Düsseldorf, Düsseldorf, Germany) for an independent samples t-test. Considering a significance level (α) of 0.05 and a statistical power (1-β) of 80%, the minimum required sample size was estimated to be 53 participants in each group. Accounting for an expected attrition rate of 10%, 58 participants were finally included in each group, resulting in a total of 116 participants.

All assessments of outcome measures, and administration of the intervention, were done under the supervision of the Principal Investigator.

Inclusion criteria for CLBP and control groups

Right-handed individuals of either sex, aged between 18 and 50 years, were included after screening. CLBP patients visiting the OPD of Orthopaedics, Neurology, and Physical Medicine and Rehabilitation were screened and recruited as per the criteria described by the National Institute of Health Pain Consortium for non-specific pathology. Cases/patients of CLBP were screened from the Clinical OPD, AIIMS Bhopal. Participants were duly informed about the purpose and details of the study and recruited from the outpatient clinics of AIIMS Bhopal after obtaining informed consent. Standardized open-access tools were used for data collection, including the VAS for pain intensity [7,11], the MODI for functional disability [12,13], the SAGE [10] for cognitive function, and ERP recordings for objective assessment of cognition. Corticomotor excitability was evaluated using TMS to measure RMT% and MEP amplitude.

The inclusion criteria for the control group are healthy, pain-free, right-handed individuals of either sex, aged between 18 and 50 years. 

Exclusion criteria for CLBP and control groups

Individuals with epilepsy, neuropathies, brain tumour, diabetes mellitus, and other psychiatric illnesses like bipolar disorder, psychosis, severe anxiety, insomnia, claustrophobia, autoimmune conditions, cardiac pacemakers, and those with contraindications to TMS - such as metallic implants, intracardiac lines, neuro-active drugs, history of seizures, major head trauma in the past six months, bullet fragments, aneurysm clips, history of opioid or substance abuse, and pregnant or lactating women - were excluded based on history taken at the time of recruitment of participants.

Outcome measured

Pain Status

Pain intensity was assessed using the VAS, which is a linear, continuous scale to quantify pain intensity. It was represented on an 11-cm-long line with two anchors: “0” = no pain and “10” = maximum pain experienced [7,11].

Disability Scores

Disability scores were assessed by the MODI, which is the gold standard for low back functional outcome. A modified disability questionnaire, designed for the Indian population by adding two questions related to bending forward and work status, and removing questions related to sexual activity and weight lifting or traveling, can help evaluate disability caused by back pain. It is a 10-item scale designed to test how back pain affects the ability to manage everyday activities. Each item has six options indicating increasing severity. The patient is asked to tick the sentence that closely reflects their daily experiences in the past. An outcome assessor assigns a score of “0” = not affected due to pain, up to “5” = extremely affected by pain. The total raw score (sum of all items) has been reported as a percentage. The cut-offs were: 0% to 20% as minimal disability; 21% to 40% indicates moderate disability; 41% to 60%, severe disability; 61% to 80% as crippled; and 81% to 100%, most likely bed-bound [12,13].

Corticomotor Excitability and Motor Threshold

Corticomotor excitability and RMT were measured using a biphasic TMS (Med-Stim MS-100 device; Medicaid Systems, Mohali, India). An angulated figure-of-eight coil was used for cortical stimulation. The TMS procedure was performed in five steps: electromyographical setup, manual localization of the tentative hotspot, confirmation of the motor hotspot, estimation of the motor threshold, and practice of recording conditions. To estimate the RMT, the coil was placed on the motor hotspot, and the standard stimulation procedure was followed. Starting at 30% maximum stimulator output, the stimulation intensity was increased in steps of 5% until clear evoked potentials appeared. The intensity was then decreased in steps of 1% until the evoked potentials disappeared (step-down), and increased again in steps of 1% (step-up) until 5 out of 10 trials yielded evoked potentials with an amplitude of at least 50 μV [7,8]. To confirm, the step-down and step-up were repeated three times. If consistent, the value was noted as the motor threshold. It was defined as “the minimum intensity at which at least 50% of trials resulted in evoked responses of more than 50 µV amplitude, keeping the target muscle at rest.”

Recording and analysis of MEPs: MEPs were recorded from the first dorsal interosseous (FDI) muscle during each TMS pulse. The peak-to-peak amplitude (in μV) of each MEP will be measured using EMG analysis software at 120% of the RMT [14,15].

Cognitive Status

The SAGE, which is a brief, self-administered cognitive screening instrument, was used to identify mild cognitive impairment from any cause. SAGE is a valid, reliable tool for detecting cognitive impairment and has comparable efficacy to the MMSE [10]. There are four forms of the SAGE test; only one test form was given. The maximum score is 22; a score of 16 or less indicates mild cognitive impairment.

Event-Related Cognitive Potential (P300)

P300 was recorded in an acoustically and electrically shielded room using the Nihon Kohden Neuropack X-1 machine (Nihon Kohden Corporation, Tokyo, Japan). Participants were tested between 9:00 AM and 11:00 AM, at least one hour after a light breakfast, and were instructed to avoid caffeine- and tannin-containing drinks for at least 10 hours before testing. They sat comfortably with their eyes closed and were asked to avoid eye movement to reduce interference. After cleaning the scalp, Ag/AgCl disc electrodes were placed on the scalp at standard positions: Fz (A11), Cz (A21), and Pz (A31), with reference electrodes attached to both mastoids, and Fpz served as the ground electrode. All necessary precautions were taken during electrode placement. The electrode-skin impedance was kept below 5 kΩ, and background noise was below 0.5 μV.

Auditory oddball paradigm: P300 was recorded using an auditory oddball task, which is the preferred modality for recording P300 waveforms. The participants listened to two types of tones through headphones. In this task, participants heard a series of sounds (standard tones) mixed with rare “oddball” tones that differed in frequency by 60 dB or intensity. The rare tones were presented randomly to avoid predictability; the improbability of the task is the main advantage in eliciting the waveform. Participants were instructed to stay attentive and press a button each time they heard a rare/oddball sound [16]. Any signals with artifacts (eye movement and muscle noise) were removed manually before averaging.

Statistics

Data were collected prospectively from 58 individuals diagnosed with CLBP and 58 age- and gender-matched healthy controls. All data were manually entered into a pre-coded Microsoft Excel spreadsheet (Microsoft® Corp., Redmond, WA, USA) and subsequently verified to ensure accuracy. The dataset was then imported into JASP software (version 0.19.3; JASP Team, University of Amsterdam, the Netherlands) for statistical analysis.

As the data did not follow a normal distribution (confirmed using the Shapiro-Wilk test), non-parametric tests were applied. Group comparisons between patients with CLBP and healthy controls were conducted using the Mann-Whitney U test for independent samples. Median values with their interquartile range (IQR) were calculated for each variable, and a p-value of < 0.01 was considered statistically significant.

## Results

The age, gender, BMI, and other variables were comparable between the CLBP and healthy control groups, and no statistically significant differences were noted. The demographic data of the CLBP patients (n = 58) and age- and gender-matched healthy controls (n = 58) are shown in Table 1.

**Table 1:** Demographic data of CLBP patients (n = 58) and healthy controls (n = 58) * indicates there is a significant difference in drug use between CLBP patients and healthy controls. CLBP: Chronic Low Back Pain

Variables	CLBP group (n = 58)	Control (n = 58)	p-value
Age, Med ± SD	36.52 ± 9.43	33.39 ± 9.30	0.075
BMI, Med ± SD	23.59 ± 3.52	22.75 ± 1.71	0.105
Gender, n (%)			
Male	34 (58%)	36 (62%)	0.849
Female	24 (12%)	22 (38%)	
Drug use, n (%)			
Yes (n, %)	13 (22%)	% 0	0.0004*
No (n, %)	45 (78%)	% 0	
Smoking and tobacco use: n (%)			
Yes (n, %)	11 (19%)	14 (24%)	0.652
No (n, %)	47 (81%)	44 (76%)	
Alcohol use: n (%)			
Yes (n, %)	9 (15%)	12 (20%)	0.480
No (n, %)	49 (58%)	46 (80%)	
Occupation, n (%)			
Home makers	14 (24%)	12 (21%)	0.794
Construction worker	5 (9%)	4 (7%)	
Employer	39 (67%)	42 (72%)	

The clinical, disability, neurophysiological, and cognitive parameters were compared between individuals with CLBP (n = 58) and age-matched healthy controls (n = 58) as shown in Table 2.

**Table 2:** Independent samples Mann-Whitney U test between CLBP cases (n = 58) and healthy controls (n = 58) The table shows a comparison of pain (VAS) score, disability index (MODI), cortical excitability (RMT, MEP), and cognitive function (SAGE, ERP-P300) between CLBP patients and healthy controls. VAS score for pain assessment, MODI score for disability assessment, neurophysiological parameters to assess excitability - RMT and MEP, SAGE, and ERP (P300) for cognitive function assessment. w: Mann-Whitney U Statistic; IQR: Interquartile Range; level of statistical significance; r = effect size (rank-biserial correlation); VAS: Visual Analogue Scale; MODI: Modified Oswestry Disability Index; RMT: Resting Motor Threshold; MEP: Motor Evoked Potential; SAGE: Self-Administered Gero-cognitive Examination; ERP: Event-Related Potential

S. No.	Parameters	CLBP cases, median (IQR)	Healthy control, median (IQR)	Test statistics (w)	p	r
1	VAS (score)	6 (2)	0 (1)	0	< 0.001	-1
2	MODI (score)	11.5 (9)	0 (1)	31.5	< 0.001	-0.98
3	RMT (%)	55 (15)	50 (5)	1050.5	< 0.001	-0.37
4	MEP (µv)	144 (218.75)	294.5 (287)	2552	< 0.001	0.51
5	SAGE (score)	17 (4)	19.5 (3)	2656.5	< 0.001	0.57
6	ERP Lat (ms)	341 (33.75)	290 (35.75)	373	< 0.001	-0.77
7	ERP Amp (µv)	7.3 (5.67)	12.45 (6.77)	2519	< 0.001	0.49

**Figure 1:**
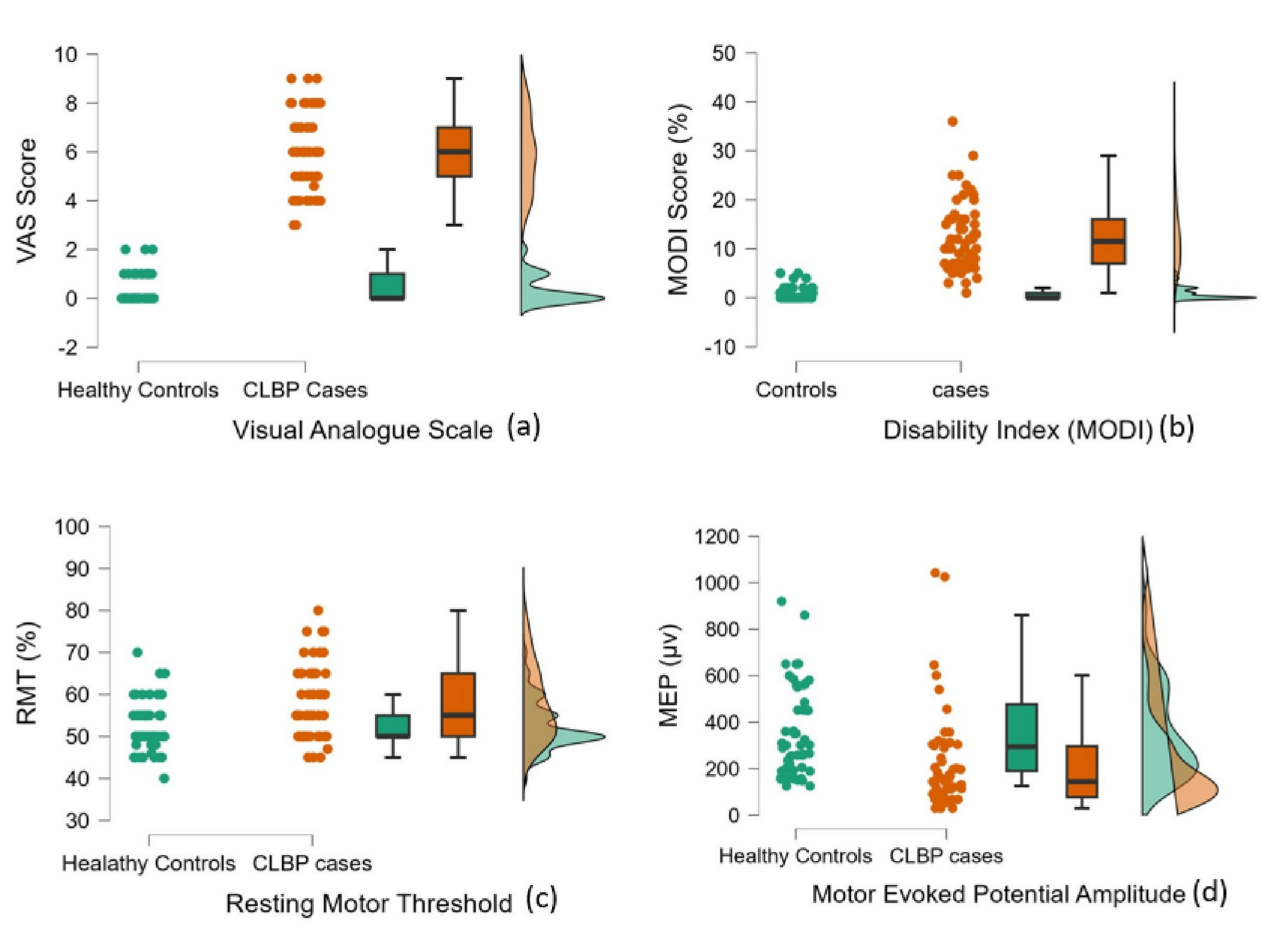
Comparison of pain (VAS) scores (a), disability (MODI) scores (b), and cortical excitability - RMT (c) and MEP (d) - between healthy controls and CLBP patients VAS score for pain assessment, MODI score for disability assessment, neurophysiological parameters to assess excitability - RMT and MEP. VAS: Visual Analogue Scale; MODI: Modified Oswestry Disability Index; RMT: Resting Motor Threshold; MEP: Motor Evoked Potential; CLBP: Chronic Low Back Pain

**Figure 2:**
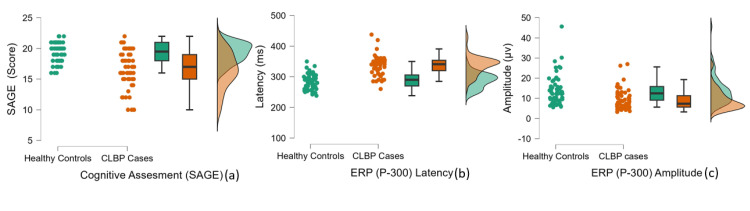
Comparison of cognitive functions assessed by SAGE (a), and ERP (P300) - latency (b) and amplitude (c), between healthy controls and CLBP patients SAGE was used for subjective, and ERP (P300) for objective, cognitive function assessment. SAGE: Self-Administered Gero-cognitive Examination; ERP: Event-Related Potential; CLBP: Chronic Low Back Pain

Figures 1-2 show the graphical representation of the comparison between CLBP patients and healthy controls across various parameters, including pain (VAS score; Figure 1a), disability (MODI score; Figure 1b), cortical excitability - RMT% (Figure 1c) and MEP (Figure 1d), and cognitive functions assessed via SAGE (Figure 2a) and ERP-P300 latency (Figure 2b) and amplitude (Figure 2c). Significant differences were observed between the groups across all variables assessed (p < 0.001).

Pain intensity, as measured by the VAS, was significantly elevated in CLBP patients (median VAS = 6, IQR = 2) compared to healthy age-matched controls (median = 0, IQR = 1) (p < 0.001; Table 2 and Figure 1a).

Disability scores on the MODI were also significantly elevated in the CLBP group (median = 11.5, IQR = 9) versus controls (median = 0, IQR = 1) (p < 0.001; Table 2 and Figure 1b).

In CLBP participants, neurophysiological parameters such as RMT% (median = 55%, IQR = 15) were higher compared to controls (median = 50%, IQR = 5). MEP amplitude (median = 144 µV, IQR = 218.75) was lower compared to controls (median = 294.5 µV, IQR = 287) (p < 0.001; Table 2 and Figures 1c-1d).

The cognitive function assessed by the SAGE score was lower in CLBP participants (median = 17, IQR = 4) relative to controls (median = 19.5, IQR = 3) (p < 0.001; Table 2 and Figure 2a).

ERP components assessing cognitive function objectively also differed significantly in the CLBP group compared to healthy controls. ERP showed prolonged P300 latency (median = 341 ms, IQR = 33.75 vs. median = 290 ms, IQR = 35.75) (p < 0.001) and reduced P300 amplitude (median = 7.3 µV, IQR = 5.67 vs. median = 12.45 µV, IQR = 6.77) (p < 0.001; Table 2 and Figures 2b-2c).

These findings suggest that there is increased pain, a higher disability index, altered corticomotor excitability, reduced cognitive performance, and overall impaired neurophysiological responses in individuals with CLBP, compared to age-matched healthy controls.

## Discussion

This study demonstrated significant differences in pain perception, corticomotor excitability, MEPs, cognitive performance, and disability levels between CLBP patients and age-matched healthy controls. Pain levels were significantly higher in CLBP patients (VAS: p < 0.001), with greater disability (MODI: p < 0.001) and decreased corticomotor excitability, as shown by significantly increased RMT% and reduced MEP amplitudes (p < 0.001). Additionally, cognitive function, assessed through SAGE scores and ERP measures (latency and amplitude), was notably impaired in CLBP patients, indicating neurocognitive alterations associated with chronic pain.

These findings highlight the multiple impacts of CLBP beyond somatic symptoms. The altered motor cortex excitability, impaired cognitive performance, and impaired perceptual learning ability seen in chronic pain patients suggest central nervous system involvement, clearly indicating that CLBP is a condition with both peripheral and central components [17]. Identifying neurophysiological and cognitive deficits in CLBP populations is crucial for developing more comprehensive and targeted rehabilitation strategies, moving beyond mere pain control to address motor and cognitive functions.

Chen et al., in their review article, stated that the relationship between chronic pain and cognitive impairment is bidirectional, and that people with chronic pain often have memory deficits, reduced attention, disorientation, executive dysfunction, and impaired emotional decision-making. They also noted that it is the persistence of pain interference (defined as the degree to which pain impairs activities of daily living), rather than pain intensity, that is associated with an increased risk of cognitive impairment. Brain gray matter atrophy may be one of the pathophysiological mechanisms underlying the development of cognitive dysfunction in patients with chronic pain [5].

The prolonged P300 latency and reduced amplitude in patients with chronic back pain, compared to healthy controls, indicate impaired cognitive processing and attentional deficits. These findings are in line with previous ERP studies showing cognitive slowing and diminished attentional engagement in conditions such as fibromyalgia, CLBP, and neuropathic pain. The prolonged latency reflects delayed stimulus evaluation, while reduced amplitude suggests lower cognitive resource allocation. Chronic pain is thought to disrupt functional connectivity in prefrontal and parietal regions involved in cognition, possibly due to neuroinflammatory processes, persistent nociceptive input, or comorbid mood and sleep disorders [18].

These cognitive impairments may hinder adherence to treatment and rehabilitation protocols, underscoring the need for integrated cognitive-motor interventions. The five mechanisms by which cognitive impairment occurs, as explained by Zhou et al., include (a) altered activity in the cortex and neural networks, (b) grey matter atrophy, (c) microglial activation and neuroinflammation, (d) comorbidities associated with CLBP, and (e) gut microbiota dysbiosis. Reduced gray matter was seen in the dorsolateral prefrontal cortex (DLPFC), medial PFC, insula, posterior cingulate cortex, cuneus, thalamus, temporal lobes, and precentral/postcentral gyrus - areas extensively linked to cognitive function [18,19]. Clinically, ERP assessment is a reliable method for objectively monitoring cognitive dysfunction in chronic pain, promoting earlier detection and multidisciplinary treatment approaches.

Our study shows that patients with chronic pain exhibited significantly lower SAGE scores compared to healthy controls, indicating a mild reduction in cognitive function among CLBP patients. This finding aligns with broader evidence from previous studies in the literature, which link chronic pain to deficits across various domains, including attention, memory, executive function, and processing speed. Psychological comorbidities, neuroinflammatory responses, increased stress hormone levels, and reduced neuroplasticity may also contribute to impaired cognitive performance [5,15,20,21].

Our findings are consistent with several earlier studies that have reported elevated MODI scores among chronic pain populations. This reinforces that chronic pain often leads to physical deconditioning, reduced mobility, and avoidance of activity due to fear of pain exacerbation (fear-avoidant behaviour). It supports the well-established relationship between chronic pain and functional limitation, as MODI is widely used as one of the standard measures for disability [22,23].

The significant reduction in corticomotor excitability (higher RMT% and lower MEPs) in CLBP patients suggests impaired neuroplastic changes, possibly reflecting disuse, altered sensory processing, or reductions in inhibitory mechanisms - suggesting disrupted cortical GABA-mediated inhibition in the primary motor cortex [23]. Dysregulation of glutamate and GABA (the brain’s main excitatory and inhibitory neurotransmitters) has been observed in various cerebral regions of individuals with chronic pain [5]. These changes may contribute to poor motor control and movement-related fear, perpetuating the pain-disability cycle. Measuring changes in RMT and MEP amplitude using TMS could function as an objective neurophysiological biomarker of chronic pain-related motor cortex dysfunction. It can help in planning tailored neuromodulatory interventions (e.g., repetitive TMS, motor imagery, or cortical excitability training) to restore normal function and guide rehabilitative measures [3,23].

Our findings align with previous studies showing decreased MEP amplitudes and increased motor thresholds in chronic pain populations, indicating motor cortex inhibition [24,25]. Furthermore, cognitive dysfunction in CLBP - particularly in attention and executive function - has been previously reported [26,27], supporting the observed lower SAGE scores and ERP alterations in our study.

Some studies show that not all CLBP patients have cognitive impairments, suggesting heterogeneity in the pathophysiology of CLBP [28]. Also, while ERP measures are sensitive to subtle changes in cognitive processing, they are influenced by multiple cortical pathways and individual variability.

One limitation is the cross-sectional design, which precludes causal inference regarding whether cognitive and corticomotor changes are causes or consequences of chronic pain. Additionally, the high standard deviation in MEP and ERP amplitude suggests substantial inter-individual variability, warranting larger sample sizes or stratification based on pain duration, intensity, or psychosocial factors. Small group size and a unicentric study design are drawbacks, although the sample size was calculated using a medium effect size and adequate power. Heterogeneity of CLBP was not explored (e.g., duration, severity, pain subtypes) due to the small sample size and will be addressed in the following longitudinal research. Another limitation is the reliance on median values without subgroup analysis (e.g., gender, chronic pain), which may obscure clinically relevant patterns. Future studies should employ longitudinal designs, multimodal imaging, and functional assessments to better understand the dynamic interplay between chronic pain, cortical excitability, and cognition.

## Conclusions

CLBP is associated with heightened pain, greater disability, reduced corticomotor excitability, and probable cognitive impairment, indicating both peripheral and central nervous system involvement. Objective neurophysiological and cognitive markers, such as RMT, MEP, and ERP parameters, may help guide targeted rehabilitation strategies. Integrating motor and cognitive interventions with pain management could address the multifaceted nature of CLBP and may improve patient outcomes. Future research should focus on longitudinal ERP changes, objective neurophysiological markers, and explore the reversibility of these motor and cognitive deficits with targeted pain interventions.
